# Montreal

**Published:** 1896-04

**Authors:** Harry H. Dell

**Affiliations:** Secretary-Treasurer


Secretary.

### MONTREAL VETERINARY MEDICAL ASSOCIATION.

A REGULAR meeting was held in the Library on Thursday, January 23, 1896, Dr. Chas. McEachran presiding.

The reading of the minutes of the last meeting and the transaction of business arising out of the same being over, the report of the Experiment Committee was called for. The Chairman of the Committee reported progress, and Messrs. McCarry and Higgins were appointed to act until the next meeting.

Mr. J. A. Ness furnished an interesting case-report and exhibited a peculiar-looking specimen from the uterus of a cow. It consisted of a band of fibrous tissue about ten inches in length, the presence of which in the uterus had given rise to symptoms simulating labor-pains. Sections had been prepared and were shown under the microscope, evidencing the fact that the growth was composed of fibrous tissue only. Dr. McEachran was of the opinion that the band was a sequel of a previous parturition in which some lacerations had occurred. He had frequently found similar growths in the vagina, apparently arising from the same cause, and which were in many cases a mechanical obstruction to delivery.

Mr. J. J. McCarry read an instructive paper on "Post-mortems," describing in an entertaining manner the methods to be followed in conducting autopsies. This led to considerable discussion on the part of the members, during which were pointed out the precautions necessary when death had occurred from a disease of a contagious nature. Dr. McEachran referred to the careless and consequently unsatisfactory manner in which post-mortems are frequently conducted, and mentioned the fact that the only English work on the subject was by Dr. Clement, of the class of '83.

The essayist for the next meeting was notified, and adjournment took place.

A WELL-ATTENDED meeting was held on Thursday evening, February 6, 1896, with the President, Dr. M. C. Baker, in the chair. The minutes of the previous meeting were read and approved. The Librarian, Mr. J. A. Ness, reported the addition to the library of several new works.

Mr. Chas. H. Higgins, on behalf of the Experiment Committee, reported progress, and requested an extension of time to furnish a report, which was granted.

Mr. J. J. McCarry reported an interesting case which had recently come under his observation. The subject was a horse which, two years ago, as the result of a boiler explosion, had been blown through the doorway of a building, but at the time was not thought to have sustained any very serious injury. Shortly afterward he developed a fistula above the point of the shoulder. A seton was inserted and the animal put to pasture. For a time recovery appeared to have taken place, but later the symptoms returned, and last December an operation resulted in the removal of a rivet-head about three-quarters of an inch in diameter from under the levator humeri, where it had probably lodged at the time of the explosion. The animal made a good recovery and is now working daily.

Mr. E. H. Morris read an excellent paper on "Hemoglobinemia," which, it was stated, is not a disease of the kidney, but under the influence of various causes a secondary nephritis may accompany it. The symptoms, pathology, sequelæ, and treatment were each in turn thoroughly described. One case, that of a mule, was instanced, that was peculiar in that the muscles of the shoulder were the ones affected. The discussion following was mostly concerned with the treatment of the disease, and after further remarks by the Chairman the meeting adjourned.

A REGULAR meeting was held on Thursday, February 20, 1896, the Honorary President, Dr. D. McEachran, occupied the chair. After the roll-call and reading of the minutes of the previous meeting the Librarian reported the addition of several new works to the library.

A committee was appointed, consisting of Dr. Dawes, Messrs. Kee and Ness, to draft resolutions on the death of Dr. Donald Campbell, '82.

Mr. F. W. Kee furnished the case-report for the evening, describing a peculiar case of "Suppurative Mediastino-pericarditis in a Horse." A prolonged discussion followed on the pathology of the case, also on the general treatment of thoracic affections.

Mr. J. A. Ness read an interesting paper on "Horse-breeding," describing the selection, care, and management of breeding-animals. The relative merits of Clydes and Percherons came first under discussion, it being the consensus of opinion that the former were better adapted to the climate of this country. Dr. D. McEachran closed the discussion by an interesting historical *résumé* of the origin of the French-Canadian horse. "They are," he stated, "the descendants of French mares and Arabs or Barbs imported from the Levant by the officers stationed in the Province during its occupation by the French." After further remarks on the subject of first impregnation the meeting adjourned.

A REGULAR meeting was held on Thursday evening, March 5, 1896. There was a good attendance of members, the Second Vice-President, Mr. E. C. Thurston, occupying the chair.

After the roll-call and the reading of the minutes of the previous meeting the Secretary read a communication from Dr. A. T. Rowat, of Honolulu, on "Paracentesis Abdominis." The technique of the operation and its indications were described by the writer in a lucid and entertaining manner. He had in his own practice proved the great utility of puncturing the intestine in cases of flatulence, and attributed the unfortunate sequelæ which some practitioners ascribe to the operation as due to lack of asepsis or of delaying the operation until the case is beyond recovery. In the discussion following Drs. C. McEachran and Martin pointed out that with proper precautions the operation was not only simple, but was safe and well warranted by the good results to be obtained. Concerning peritonitis in the horse as a sequela, it was thought that the peritoneum of that animal is not so liable to inflammatory lesions following trauma or surgical interference as is generally supposed, and many instances were adduced in support of this view. A motion by Mr. Kee was unanimously carried, "that the Secretary be instructed to convey to Dr. Rowat the thanks of the Association for his communication, which is most highly appreciated, evidencing on the part of the writer the interest he maintains in the welfare of the Association."

Mr. C. H. Higgins read a case-report of "Traumatic Ventral Hernia, with Rupture of the Udder, in a Cow," that evoked further discussion on peritonitis, during which Mr. Higgins pointed out the great field, as yet untouched, for research concerning the contents of the intestinal tract of the various domestic animals from a bacteriological point of view.

Mr. S. C. Richards presented an admirably prepared paper on "Acute Specific Pleurisy in the Horse." He described the clinical history and post-mortem changes found in an animal which he recently had under his observation. In the discussion following Dr. Martin stated that, in human practice, a bacteriological examination of the pleuritic effusion was an invaluable aid in arriving at a correct diagnosis of the pathological changes present, and why should the same not be true of veterinary practice?

Mr. Higgins reported, on behalf of the Experiment Committee, that the dog from which the spleen had been removed six weeks previously had been destroyed. Beyond a chronic peritonitis at the site of the operation no other abnormalities were noted. There was no glandular enlargement of any description. Blood-counts were not made.

Mr. J. H. Patterson promised a paper on Colic for the next meeting, and adjournment took place.

HARRY H. DELL,

Secretary-Treasurer.

